# Minding morality: ethical artificial societies for public policy modeling

**DOI:** 10.1007/s00146-020-01028-5

**Published:** 2020-08-07

**Authors:** Saikou Y. Diallo, F. LeRon Shults, Wesley J. Wildman

**Affiliations:** 1 Virginia Modeling, Simulation and Analysis Center, Old Dominion University, 1030 University Blvd., Suffolk, VA 23435 USA; 2 Center for Modeling Social Systems at NORCE, and Institute for Global Development and Social Planning, The University of Agder, Universitetsveien 19, 4633 Kristiansand, Norway; 3 Center for Mind and Culture, and Boston University, 745 Commonwealth Avenue, Boston, MA 02215 USA

**Keywords:** Multi-agent artificial intelligence, Social simulation, Public policy, Ethics, Morality, Cultural norms

## Abstract

Public policies are designed to have an impact on particular societies, yet policy-oriented computer models and simulations often focus more on articulating the policies to be applied than on realistically rendering the cultural dynamics of the target society. This approach can lead to policy assessments that ignore crucial social contextual factors. For example, by leaving out distinctive moral and normative dimensions of cultural contexts in artificial societies, estimations of downstream policy effectiveness fail to account for dynamics that are fundamental in human life and central to many public policy challenges. In this paper, we supply evidence that incorporating morally salient dimensions of a culture is critically important for producing relevant and accurate evaluations of social policy when using multi-agent artificial intelligence models and simulations.

## Introduction

Testing complex policies in the real world is difficult due to ethical considerations, cost of evaluation, and challenges in generalizing test outcomes. It is understandable, therefore, that policy professionals would turn to computational policy modeling as an ethical and affordable way of generating cost–benefit estimates of policy proposals before they are implemented. To keep such models manageable and affordable, policy modelers naturally seek to make reasonable simplifications of formidably intricate cultural contexts, knowing that there is always a price to be paid for such simplifications and abstractions. Good policy models aim to strike the balance between complexity and abstraction in such a way as to optimize accuracy in projections related to the specific policy in question (Edmonds and Moss 2004; Jager 2017). But it is difficult to generate concrete estimates of the price paid for a decision to abstract from any given dimension of cultural life. We contend that including specific morally laden aspects of a society (e.g. marriage rituals, patterns of interpersonal contacts, behavioral prohibitions) can drastically alter the estimated impact of a policy, which implies that policy models lacking those distinctive moral features are limited in their relevance and accuracy. As a guide to future work in policy simulation, we identify baseline aspects of an artificial society that are needed to provide more accurate evaluations of the impact of almost any social policy in a complex social system.

It is important to note that our attempt to overcome this particular limitation in most current approaches to public policy modeling (the failure to mind morality) is not meant to obscure the many other limitations that are inherent to this methodology. As the common adage goes: “all models are wrong, but some are useful.” The goal is to render one’s model as useful as possible while acknowledging the ways in which it is wrong, as well as its epistemological and hermeneutical limitations (Tolk et al. 2018; Tolk 2019). In this context, our goal is not to defend the assumptions or validate the specific outcomes of the particular simulation experiments outlined below but to point out the extent to which including (or failing to include) morally salient features within an artificial society impacts the policy relevance of any of its outcomes. This argument will become increasingly important as simulation experiments within artificial societies are used to address societal challenges such as the COVID-19 pandemic (Squazzoni et al. 2020) and conflict exacerbated by climate change (Shults and Wildman 2020), the effects of which vary significantly across diverse cultural contexts.

## State of the art review

The systems in relation to which public policies must be proposed, analyzed, implemented, and evaluated are exceedingly complex. All too often policy professionals are faced with “wicked problems” within these systems, in the sense that some “solutions” can cause unexpected perturbations that make things worse. As the human population and consumption of resources continue to grow, so does the urgency of the need to develop new ways to address this sort of problem (Cliquet and Avramov 2018).

As an example, consider Dengvaxia, which won FDA approval in 2019 (US Food and Drug Administration 2019) as a vaccine to prevent recurrence of Dengue Fever in children aged 9–16 who had already been infected once. Dengue Fever is the most prevalent mosquito-borne viral disease, directly affecting one-third of the world’s population. Preventing a second infection is the key to avoiding the worst effects of the Dengue virus. Unfortunately, when administered before prior infection, the vaccine acts like a first infection and can make a second infection more dangerous. This was an unexpected result and led to the deaths of a small number of children in the Philippines. For this reason, the approved deployment of Dengvaxia *after prior infection*, despite all the good it is doing, also threw fuel on the fire of anti-vaccination sentiment. Anti-vaxxers routinely cite the case, and this has directly contributed to millions of parents refusing vaccines for their children even when those vaccines are known to have extremely rare or no side effects, leading in turn to new outbreaks of deadly infectious diseases. This is an unintended side effect of a well-intended policy intervention with highly non-linear amplification in an unexpected direction. It is precisely this concern about the possibility of disastrous unintended consequences that leads experts to be very cautious about the timeline for a vaccine to combat COVID-19, the disease caused by the SARS-CoV-2 virus. Meanwhile, political pressures on non-expert policy makers render them all too ready to risk making past mistakes all over again.

In recent years, an increasing number of computational social scientists have risen to the wicked-problem challenge. In 2012, several leading scholars in this field offered a “manifesto of computational social science,” identifying tools for dealing with the Big Problems of society. Developments in this field, they argued,

will make it possible to model and simulate social processes on a global scale, allowing us to take full account of the long-distance interdependencies that characterise today’s heavily interconnected world. The output of these simulations will be used to support policy makers in their decision making, to enable them to efficiently and effectively identify optimal paths for our society. Similarly, open access to these large-scale simulations will support individuals in their evaluation of different policy options in the light of their personal needs and goals, greatly enhancing citizen participation in this decision process. These developments together open the doors to a much safer, more sustainable and fairer global society (Conte et al. 2012).

We have not yet passed through those doors. Despite the intense interest in promoting wide-scale use of computational methodologies for modeling and simulating policy, there has not yet been a breakthrough.

Nevertheless, computational social science is making progress. It has not gone unnoticed within the field that complexity science directly bears on policy considerations when it comes to developing realistic agent-based models for social simulation. There are at least two different ways that complexity theory can help: “First, it can help provide representational models that might be used to constrain the range of strategies under consideration and, second, can help inform second-order considerations concerning the ways in which policy might be developed and/or adopted—the policy adaptation process itself” (Jager and Edmonds 2015, 64). The productivity of linking social simulation and complexity science is also evidenced in many contributions to *Simulating Social Complexity: A Handbook*, which provides philosophical and methodological reflection on the process as well as multiple examples (Edmonds and Meyer 2017).

Computational modeling based on research in complexity science has been applied to a host of policy-relevant issues including global migration, intergroup conflict, strategies of counterinsurgency, and international development aid (Wilson 2016; Neumann 2014; Pechenkina and Bennett 2017). The experimental results and applications of one of the most well-known, the “Simulating Knowledge in Innovative Networks” (SKIN) model, are reported in *Joining Complexity Science and Social Simulation for Innovation Policy* (Ahrweiler et al. 2016)*.* A recent article in the *Journal for Artificial Societies and Social Simulation* reviewed several other examples of state-of-the-art approaches and furnished reflections on the practice of applying these techniques to policy making. Despite the difficulties, the authors point out notable successes and stress the importance of learning from past mistakes to develop better policy models. They conclude that “where the costs or risks associated with a policy change are high, and the context is complex, it is not only common sense to use policy modeling to inform decision making, but it would be *unethical not to*” (Gilbert et al. 2018, 13).

We agree. However, experts in these fields are facing limitations as they press toward ever more policy-relevant approaches to social simulation. Scholars are increasingly recognizing the importance of engaging such stakeholders early in the development process and clearly explaining the complexity of non-equilibrium systems such as our social worlds, which means that risk cannot completely be mitigated in the “art of policy making” (Rosewell 2017). Another challenge is developing user-friendly interfaces that policy professionals find useful, and this may involve gamification or story-telling within the policy-modeling process (Desai 2012).

A deep challenge is producing artificial societies sufficiently realistic that policy professionals find them plausible and feasible for exploring the real-world complexities of social life. These experts need to be convinced that the simulations capture what is needed to model the social realities and proposed policies under consideration. We argue that ethics and social norms are so central to real societies that ignoring how they affect the interactions among simulated agents effectively invalidates a computational policy model. We offer a novel solution to mitigate this problem.

Broadly speaking, computational social science has generated two major approaches to studying and simulating individual morality and social norms in artificial societies.

The first, and oldest, is game-theoretic approaches, such as iterative prisoner’s dilemma games. Although they are relatively simple models, even game-theoretic artificial societies can address the issue of norms, because agents have different strategies for defecting or cooperating (moral concerns, surely) that affect their interactions with other agents (Binmore 1994). However, this sort of model typically assumes agents are actuated solely by rational reflection on self-interest, and thus has been heavily criticized for not capturing the complexity of decision-making and the bounded rationality of actual human agents. Such models do not capture the nuances of ethical behavior, only the abstract decision to defect or cooperate. Still, evolutionary game-theoretic models can provide insight into which strategies are likely to “win” over time, and have been applied to a variety of policy-relevant issues, especially in economics (Caldas and Coelho 1999; Hamill and Gilbert 2015). Game-theory models are helpful when it comes to simulating the emergence of cooperation and the role that dynamics such as reputation management play in shaping norms (Corten 2014). Some scholars have even attempted a sort of “experimental ethics,” using game-theoretic approaches to test the adaptive role of (im)moral behaviors in various evolutionary contexts (Mascaro 2010).

The second approach utilizes multi-agent artificial intelligence (MAAI) strategies to construct more complex agent architectures for studying and simulating norms. These agent-based models have more complex cognitive architectures, social network links, and environmental variables than game theoretic models, and thus, they can shed more light on the (in)famous problem of linking macro- and micro-level dynamics in social science. In these approaches, however, the challenge is to simulate recursive interactions between inter-agent and intra-agent processes. There is a robust discussion in the field of computational social science about how to model something as complex as human norms, which are embedded within a wide variety of contexts as complex as human cultures themselves. Moreover, policy modeling with MAAI cannot be complete without also accounting for the cognitive dynamics that play a role in decision making, norm diffusion, etc. (Dignum et al. 2010; Neumann 2012; Verhagen 2001). The cognitive and psychological realism of simulated agents in MAAI models has increased rapidly in recent years, which has improved their explanatory and forecasting power in policy-relevant domains such as immigrant integration (Gore et al. 2019), the mitigation of intergroup conflict (Shults et al. 2018a, b, c), and the role of education in secularization (Gore et al. 2018).

There exist several article-length reviews of normative agent architectures (Luck et al. 2013), normative multiagent systems (Mahmoud et al. 2014), and simulation models of norms (Neumann 2012), as well as book-length analyses and reviews of the complexity of modeling norms (Xenitidou and Edmonds 2014; Elsenbroich and Gilbert 2014). Significant efforts have been made to simulate the emergence of norms (Savarimuthu et al. 2008; Frantz et al. 2014), mechanisms involved in norm compliance (Andrighetto and Conte 2012), the internalization or “immergence” of norms (Conte et al. 2014), as well as the spread of different types of norms (Merdes 2017; Flache et al. 2017).

Some scholars in the field of computational social science have even moved toward simulating culture, encroaching on the territory of anthropologists and others interested in deep description of human reality. Can computational models contribute to understanding and interpretation, not merely explanation and prediction? There is a growing number of attempts to answer this question positively (Suarez and Sancho 2010; Lotzmann and Neumann 2017). Such models are getting increasingly complex, including physical, individual, functional, structural, social, normative, and informational dimensions. Moreover, scholars are increasingly attending to the crucial role played by context in social simulation and policy modeling (e.g. Dignum and Dignum 2014). For example, one set of agent-based simulation experiments demonstrated the way in which the uptake of policies in different settings was differentially shaped by values and norm compliance within distinct cultures (Dechesne et al. 2013).

These remarkable efforts to wed social simulation to policy modeling via complexity science, while vital, are relatively ad hoc, starting with a policy in mind, then constructing an appropriate agent architecture or game-theoretic experiment, with few established best-practice guides. In particular, an evidence-based appreciation for whether and how to include the moral and ethical dimensions of societies is lacking. We propose a more rigorous approach. To our knowledge, prior to our efforts described below no one has taken all four of the following steps in this order: (1) construct an artificial society that carefully attends to the role of norms in shaping agent decisions and interactions, (2) validate that artificial society to show that it is capable of simulating dynamics in the real world, (3) implement a particular policy within that artificial society to see what changes, and (4) validate the model again to determine if the changes in the artificial society correspond to changes in real-world societies. By doing this, it is possible to develop concrete estimates of the price paid for omitting consideration of the normative aspects of human social life.

For example, consider the non-pharmaceutical interventions (NPIs) recommended or imposed on populations in the wake of the SARS-CoV-2 pandemic. The primary aim of NPIs is to prevent overwhelming the capacity of the medical system, thereby preventing avoidable deaths; their secondary aim is to minimize infections of vulnerable populations. Numerous epidemiological models depicted the effect of NPIs on reducing infections by limiting contact rates. Sometimes experts would openly acknowledge that compliance with NPI rules is critical and everyone knew that it is an important human factor. But compliance and non-compliance involve a complex set of values and norms, and complex flows of information and social networks, so they were not included in the epidemiological models, despite the fact that compliance is the single most important factor in the effectiveness of NPIs and capable all by itself of vitiating a national plan for public health. There is a price paid for omitting consideration of human social norms and moral perspectives.

Taking relevant account of the normative aspects of human social life is a tough nut to crack, but it is a necessary (though surely not a sufficient) condition for developing artificial societies that are sufficiently complex to test policies in relevant ways. Human social life is inextricably ethical. Moral norms shape everything we do. Insufficiently accounting for this reality is problematic not only for philosophical reasons but also, as we show in the next section, for predicting the effects of policy proposals. In conformity with the study of social norms within computational social science that we traced in the literature review, we argue that the role of ethical norms is so central to any society that they must be incorporated (at least minimally) for any model of a society to be relevant for public-policy evaluation. It is not simply that modeling morality in an artificial society is a helpful add-on in some cases; rather, norms should *always* be expressed in models involving policy evaluation. The goal of the next section is to show the statistically significant difference between runs that do and do not account for norms.

## Investigating the effects of norms on social simulation

Here, we expand a previously developed agent-based model with a majority group and a minority group living in a western city: the “Artificial Society Analytics Platform” (ASAP). A detailed implementation of ASAP and supplemental online materials with discussions on how it is validated are published elsewhere (Shults et al. 2020; Puga-Gonzalez et al. 2019). Agents in the model can marry and have children who inherit traits from their parents and are influenced by their experiences and environment. Over time, older agents die out and children grow up, get educated, and become adults, looking for employment and marriage partners, thus changing the population landscape. Adults and children interact weekly with their family, neighborhood, and co-workers (for employed adults). The outcome of the model is that minority agents are integrated, assimilated, or alienated and majority agents are hostile or welcoming towards minorities. The goal of the model is to explore policies that favor certain outcomes (e.g. integration in a harmonized country) over others (e.g. inter-group alienation or balkanization). We use this model here to study the policy effects of norms governing the number and types of interactions between agents. We focus on norms governing inter-agent contacts, because in this artificial society, personal encounters between agents drive how agents form attitudes, change opinions, make decisions, and take action.

ASAP was developed in collaboration with subject-matter experts and its architecture incorporated insights from sociological theories relevant for understanding immigrant integration. In this context, however, our focus is not on defending the assumptions of the model or on validating the specific outcomes of the following simulations, but on demonstrating the statistically significant differences that emerge when “minding morality.”

We conduct two experiments to study the effects of accounting for norms on policy goals. In the first experiment, we explore the degree to which the addition of norms governing inter-personal interactions leads to significant differences in inter-agent contact. In the second experiment, we investigate the degree to which including norms significantly changes outcome measures related to minority integration. Supplementary materials including raw data and source code are provided here: https://drive.google.com/drive/u/1/folders/1REMHl_tYssHhgmEZj4COhTutjrOmhbXS.

### Experiment #1: impact of norms on agent interactions

The first experiment collects data from the model running under three configurations, as follows.

- Baseline: The baseline experiment does not impose norms on the interactions between groups, meaning agents can interact regardless of age, gender, or membership in the majority or minority groups. It is important to note that the baseline model is how agent-based models of populations normally run.
- Normative: The normative experiment imposes the norms of an open western society. The norms govern interactions between (1) adults and children and (2) males and females of (3) the majority and minority groups.
- Restricted normative: The restricted normative experiment imposes norms of a more conservative (semi-open, semi-closed) western society. For example, interactions of children and females with adult majority and minority males are highly restricted.

For each configuration, we fix initial conditions and vary only the likelihood that two agents will interact given their group, age, and gender when an encounter occurs in a neighborhood or offline setting (i.e., any interaction that is not workplace-related, neighborhood-related, family-related, or online). Offline settings are meant to capture interactions that occur at gatherings, sporting events, or other social settings where there is a mix of people, only some of whom know each other. We run each configuration for thirty years with 30 replications and collect data annually.

Table 1 shows the likelihood that an interaction could occur between agents in the normative configurations. A humanities scholar expert in identifying social norms generated the estimates based on experience, the literature on social norms in a western country, and discussions with other scholars. The numbers represent relative likelihoods; a different group of scholars could use different numbers yet, if the relative scale were maintained, the results will hold.

**Table 1:** Normative probability table for interactions among eight categories of agents

	Adult male majority (AMMj)	Adult female majority (AFMj)	Child male majority (CMMj)	Child female majority (CFMj)	Adult male minority (AMMi)	Adult female minority (AFMi)	Child male majority (CMMj)	Child female minority (CFMi)
Likelihood of interaction in a neighborhood setting (from min 0 to max 1)								
Adult male majority (AMMj)	1	1	1	0.6	1	0.5	0.7	0
Adult female majority (AFMj)	1	1	0.2	0.8	1	1	0.6	0.6
Child male majority (CMMj)	0.45	0.1	1	1	1	0	1	0.5
Child female majority (CMMj)	0.5	0.35	1	1	0.6	0.8	1	1
Adult male minority (CMMj)	1	1	0.3	0.1	1	1	0.9	0.7
Adult female minority (CMMj)	1	1	0.2	0.7	1	1	0.7	0.9
Child male majority (CMMj)	0.4	0.6	0.7	1	0.9	0.7	1	0.9
Child female minority (CMMj)	0.1	0.9	0.3	1	0.5	0.9	0.9	1
Likelihood of interaction in an offline social setting (from min 0 to max 1)								
Adult male majority (AMMj)	1	1	1	0.9	1	0.6	0.7	0.4
Adult female majority (AFMj)	1	1	1	1	1	1	0.8	0.9
Child male majority (CMMj)	0.5	0.45	1	1	1	0.8	1	0.9
Child female majority (CMMj)	0.45	0.5	1	1	1	1	1	1
Adult male minority (CMMj)	1	1	1	1	1	1	1	1
Adult female minority (CMMj)	1	1	0.9	1	1	1	1	1
Child male majority (CMMj)	0.9	1	1	1	1	1	1	1
Child female minority (CMMj)	0.8	1	1	1	1	1	1	1

The values in Table 1 represent the normative expectations of the society. For instance, a value of 1 in the first cell means that it is completely acceptable for two adult males from the majority group to interact when they meet in the neighborhood while shopping or at the park. On the other hand, a value of zero in the last column of the same row means that it is unacceptable for adult males in the majority group to interact with female children of the minority group. When norms are not applied (the baseline condition) all cells take on a value of 1 and interactions are driven solely by location and chance. In the restricted norm version of this table, every cell that is not 1 is divided by two. For instance, the majority adult male/child female (AMMj to CMMj) value for interactions in the neighborhood setting moves from 0.5 to 0.25 (0.5/2). This systematic halving maintains the ratio of interaction values while further restricting the permissibility of contact.

**Fig. 1:**
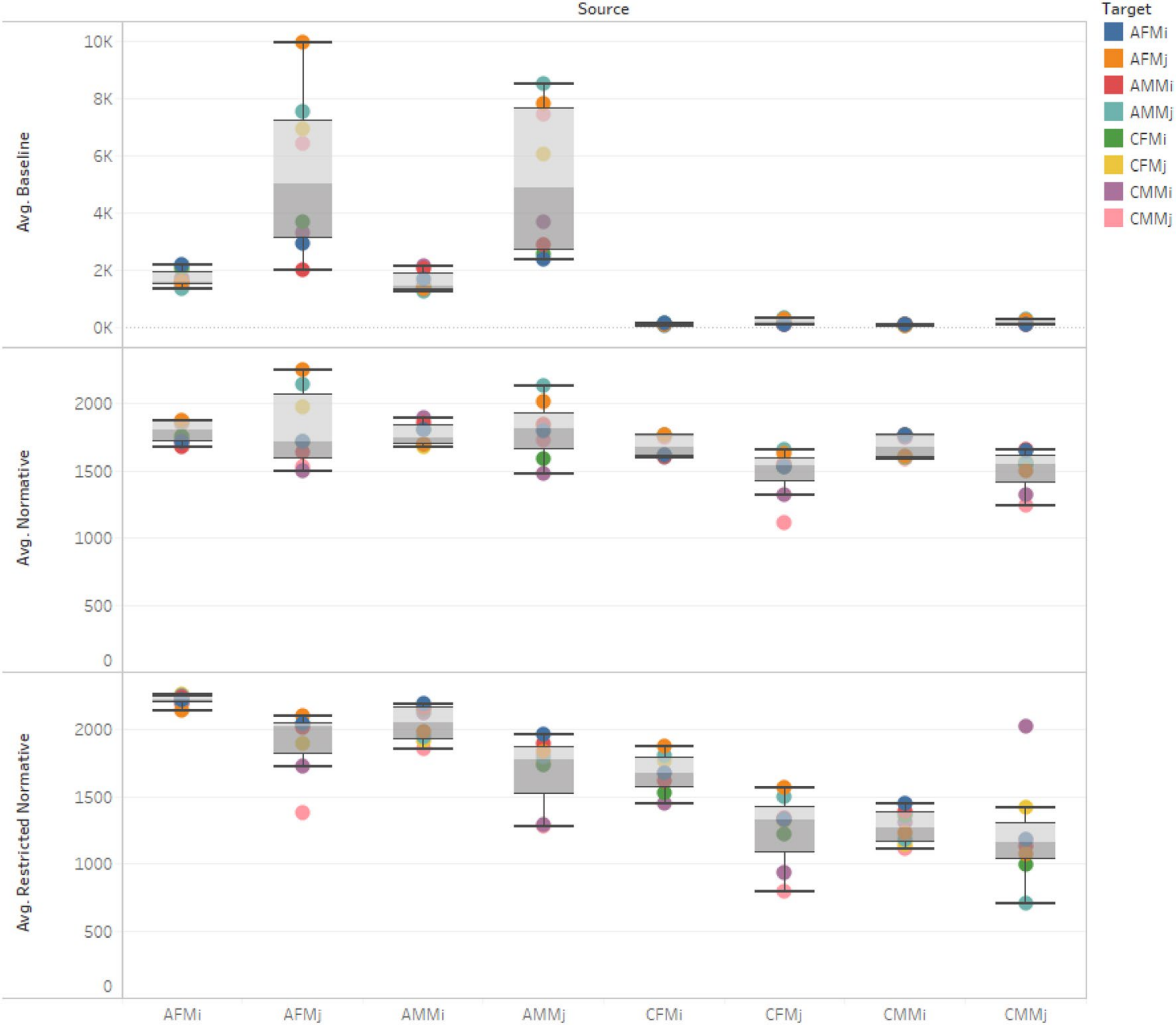
Effects of norms on amount and type of interactions between “Source” and “Target” agents for the three configurations: Baseline (top), Normative (middle), and Restricted normative (bottom). The graph shows that the number of interactions between majority and minority subgroups is significantly altered by the introduction of norms. The important aspect of this graph is the how each configuration is different from the others in terms of group to group interactions. In effect, we are dealing with three completely different societies

Figure 1 illustrates how the introduction of norms affects the inter-agent contacts across the three conditions over 30 years of interactions. Since we are sampling from normal distributions to generate the majority and minority population characteristics, we use a *t* test to compare means of the total number of interactions of each type in each configuration (95% confidence level). It is important to note that we are comparing simulations that are initialized and executed using random numbers generated by the simulation engine. There is a statistically significant difference between baseline and normative configurations of the model (*p* < 0.01). The effects are predictable. For example, since the model does not allow adult males in the majority to interact with female children in the minority, those children are only influenced by males in the minority and females and children in both groups. As a result, the overwhelming presence of males of the majority group does not overtake the interaction space, which means that there is potential for localized and isolated effects such as alienation for teenagers and integration for adults of the same group.

Furthermore, the means comparison test reveals that there is no statistically significant difference between normative and restrictive normative configurations of the model (*p* = 0.9) with respect to the total number of interactions. *This finding demonstrates how important it is to include norms in artificial societies*, even if their actual values are subjective and do not precisely match the society being modeled. In other words, even though the difference between norms may itself not always be important, the difference between including norms (of any kind) and not including norms at all is very large.

### Experiment #2: impact of norms on policy interpretations

The second experiment raises the critical question about the effect on integration-related policy measures of the three conditions under investigation. We hypothesize that testing policy in an un-normed model as opposed to a normed model will significantly alter results. This experiment employs three conditions: The baseline and normative conditions from above, together with an inclusion condition that simulates a simple policy to encourage maximum interaction between children to promote integration at an early age. This amounts to changing Table 1 by inserting a 1 for every child-to-child interaction.

We ran each configuration for 30 years seeking a 95% confidence level for the value of two integration-related measures: “Shared Norms” and “Outgroup Suspicion.” We present data on the resulting distribution of those two outcome measures in Table 2. The two integration-related measured have the following meaning.

- Shared Norms is an indication of *cultural integration*, which refers to Shared Norms and values, shared cultural capital, and a shared pluralistic attitude to religious and cultural diversity. In this model, it acts as a reasonable proxy measure for how united the majority and minority populations are within the overall population. Higher levels of Shared Norms indicate that integration policies are probably working.
- Outgroup Suspicion is a measure of *social integration*, which refers to people interacting in personal and impersonal ways, from fleeting commercial relationships to intimate personal relationships to online relationships. Lower and decreasing levels of suspicion indicate that integration policies are probably working.

**Table 2:** Results from experiment #2—comparing means of selected integration-relevant measures for the three conditions

Condition	Integration-related variable	Mean	Min	Max	SD
Baseline	Shared norms—all	0.52	0.50	0.56	0.01
Baseline	Shared norms—majority	0.50	0.47	0.53	0.01
Baseline	Outgroup suspicion—all	0.29	0.25	0.33	0.02
Baseline	Outgroup suspicion—minority	0.61	0.54	0.68	0.04
Normative	Shared norms—all	0.49	0.47	0.52	0.01
Normative	Shared norms—majority	0.50	0.47	0.52	0.01
Normative	Outgroup suspicion—all	0.18	0.15	0.21	0.01
Normative	Outgroup suspicion—minority	0.35	0.26	0.40	0.03
Inclusion	Shared norms—all	0.50	0.47	0.53	0.01
Inclusion	Shared norms—majority	0.50	0.47	0.53	0.01
Inclusion	Outgroup suspicion—all	0.18	0.16	0.21	0.01
Inclusion	Outgroup suspicion—minority	0.35	0.29	0.43	0.03

Ideally, an effective pro-integration policy would lower Outgroup Suspicion, while increasing Shared Norms. Values of Shared Norms and Outgroup Suspicion are initialized at 0.5 in every condition and their values at the end of 30 years are analyzed in Table 2.

Consider the inclusion (normed) condition relative to the baseline (un-normed) condition. In the normed condition, Shared Norms for the entire population is 3.8% lower (0.52 versus 0.50). Meanwhile, Outgroup Suspicion is 37.9% lower in the normed condition for the entire population (0.29 versus 0.18) and 42.6% lower for the Minority group (0.61 versus 0.35). This indicates that the policy greatly reduces suspicion between the two groups even though it leaves Shared Norms almost unchanged.

If the criteria for success of the Inclusion policy are increasing Shared Norms AND reducing Outgroup Suspicion, we would regard the policy as only partially successful. However, if the criterion for policy success is increasing Shared Norms OR reducing Outgroup Suspicion, we would regard the Inclusion policy as wildly successful. That is what the policy simulation would tell us by comparing the inclusion condition against baseline, at any rate. But now, compare the normative condition with the inclusion condition. The means for the key integration-related variables are virtually identical; indeed, a *t* test indicates no significant difference. It follows that the significant difference from baseline is delivered not by the Inclusion policy but merely by accounting for social norms that govern inter-personal interactions.

## Conclusion

The conclusion here is unmistakable: *we cannot trust the findings of policy simulations when the artificial society in which they are being tested does not take account of relevant social norms*.

It is important to note that this problem cannot be resolved merely by more adequate validation. The problematic assumption we are challenging is that moral norms are not important enough for modelers to include in their artificial societies. We recognize the technical challenges involved (e.g. trackability, computational tractability, memory demands, etc.). However, we have shown that relevant social norms should be included if one’s goal is to render social simulations relevant to sound assessment of policy initiatives. This has implications for ongoing conversations about the ethical assumptions and implications in the development and deployment of policy-relevant multi-agent artificial intelligence models (Shults et al. 2018c; Shults and Wildman 2019). Social norms, and more generally, the moral and ethical dimensions of human social life, are more than optional considerations for computational social scientists; they are critical for the relevance of policy simulation.
